# Student perspectives on integrating basic and clinical dental sciences: insights from three dental schools in Norway

**DOI:** 10.3389/fdmed.2026.1746468

**Published:** 2026-01-26

**Authors:** Anna Tostrup Kristensen, Noora Helene Thune, Qalbi Khan, Tor Paaske Utheim, Hugo Lewi Hammer, Amer Sehic

**Affiliations:** 1 Institute of Oral Biology, Faculty of Dentistry, University of Oslo, Oslo, Norway; 2 Department of Medical Biochemistry, Oslo University Hospital, Oslo, Norway; 3 Inland Norway University of Applied Sciences, Elverum, Norway; 4 Department of Plastic and Reconstructive Surgery, Oslo University Hospital, Oslo, Norway; 5 Department of Computer Science, Faculty of Technology, Art and Design, Oslo Metropolitan University, Oslo, Norway

**Keywords:** basic science, clinical relevance, curriculum, dental education, integration (horizontal/vertical)

## Abstract

**Background:**

A strong foundation in basic medical sciences is essential for dental education, yet their integration with clinical training remains debated. Traditional curricula often front-load biomedical content in the early years, separated from clinical practice, which may affect student motivation and perceptions of relevance. Norway's three dental schools differ in curricular design, providing an opportunity to explore how structure shapes student experiences.

**Materials and methods:**

A cross-sectional survey study was conducted by inviting 157 final-year dental students from the University of Oslo (UiO), the University of Bergen (UiB), and UiT The Arctic University of Norway to complete an anonymous online questionnaire. Ninety-eight students responded (57% in Oslo, 59% in Bergen, 75% in Tromsø), of which 94 were included in the final analysis. The questionnaire addressed expectations before admission, timing and integration of biomedical sciences, clinical relevance, and motivational impact. Data were analyzed using ordinal logistic regression and Fisher's exact test, with significance set at *p* < 0.05.

**Results:**

Over 80% of students across institutions reported being aware of the importance of biomedical sciences before admission. All three schools concentrated teaching in the first two years, but significant institutional differences emerged regarding integration in later years (*p* < 0.001). At UiO, 70% reported that extensive early teaching negatively affected their motivation, compared with fewer than 20% at UiB and UiT (*p* < 0.0001). Similarly, 65% of UiO students disagreed that clinical supervisors reinforced biomedical relevance, while students at UiB and UiT were more likely to report neutral or positive experiences (*p* < 0.001).

**Conclusion:**

Curriculum structure plays an important role in shaping students' motivation and perceptions of biomedical relevance. Programs with limited linkage between preclinical and clinical teaching may hinder students' ability to apply foundational knowledge, whereas earlier integration appears to support stronger engagement. Strengthening vertical integration may therefore enhance both clinical competence and perceived relevance.

## Introduction

There is broad consensus that a solid foundation in basic medical sciences is essential for the education of future dentists (1, 2). As healthcare systems evolve and patient cases become increasingly complex (3), dental professionals are expected to manage not only oral conditions but also broader systemic health issues. This is relevant among aging populations with multiple comorbidities and extensive pharmacological regimens (4). These demographic and clinical shifts underscore the importance of comprehensive medical knowledge in dental practice and highlight the necessity for dentists to possess a robust understanding of general health to deliver safe, effective, and holistic care (5).

Despite this acknowledged need, the way basic medical sciences are integrated into dental education remains a subject of debate (6), and the conceptualization, implementation, and experience of “integration” vary substantially across dental schools. In this study, we define integration using established educational terminology: *horizontal integration* refers to linking basic sciences with related subjects taught concurrently, whereas *vertical integration* denotes the connection between preclinical sciences and later clinical training, including the reinforcement of foundational concepts during patient-based learning. In many institutions, the early years of dental training are dominated by theoretical instruction in foundational subjects such as anatomy, physiology, immunology, and pathology, often delivered alongside medical students (7). While this approach provides a shared scientific base, it is frequently taught in isolation from clinical context (8). As a result, students may struggle to apply this knowledge later in their training, when it becomes most relevant. This separation between preclinical theory and clinical practice has been associated with reduced retention of key concepts (9), diminished engagement, and a limited perception of relevance (10, 11). Moreover, previous studies suggest that poorly integrated curricula can adversely affect students' professional identity and motivation, potentially leading to dissatisfaction, uncertainty, or program attrition (8).

The central pedagogical challenge, therefore, lies in creating meaningful integration between basic medical sciences and clinical dental education. Many students report difficulty linking abstract theoretical knowledge to real-life clinical scenarios, an issue that can impede learning outcomes and reduce clinical confidence (7, 12). This raises several critical considerations, including which aspects of medical knowledge are essential for dental professionals, when this content should be introduced to ensure long-term retention and applicability, and how it can be delivered to make its clinical value clear and enduring. Traditionally, dental education has been organized into two distinct phases: a preclinical phase (typically the first two years) focusing on basic sciences, followed by a clinical phase (years to three to five) dedicated to patient care. There is growing international interest in bridging this divide by embedding clinical relevance into basic science education and reinforcing basic medical concepts during clinical training, which has been shown to enhance engagement, strengthen understanding, and improve long-term knowledge application (13). Although dental education research has explored curriculum integration from the student perspective to some degree (14–16), but few have examined European programs, and no study has compared all three Norwegian dental schools.

As the primary recipients of educational reform, students provide invaluable insights, and understanding their experiences of integrating theory and practice is essential for developing curricula that are both pedagogically effective and professionally meaningful. This study seeks to address this gap by investigating how final-year dental students at Norway's three dental schools in Oslo, Bergen, and Tromsø perceive the integration of basic medical sciences and clinical training. These institutions differ somewhat in how they structure and integrate the basic and clinical components of their curricula, providing a valuable basis for comparison. In this context, integration is broadly defined as the consistent presence of clinical relevance in preclinical teaching and the incorporation of basic medical concepts in clinical education and patient care. However, the study deliberately left the definition of “integration” open-ended in its survey design to capture the full breadth of student interpretation and experience.

To address these questions, the present study examines how final-year dental students across Norway perceive the integration of basic and clinical sciences, with particular attention to when and how biomedical relevance is introduced in the curriculum. Because the three national dental schools employ different curricular structures, the study also explores how these institutional contexts may shape students' experiences of integration, clinical relevance, and pedagogical continuity. By combining national comparative data with an in-depth analysis of students' perceptions, this study aims to provide context-specific evidence that can inform ongoing curriculum development and contribute to broader discussions on integration in dental education.

## Materials and methods

### Study design and population

**Figure 1:**
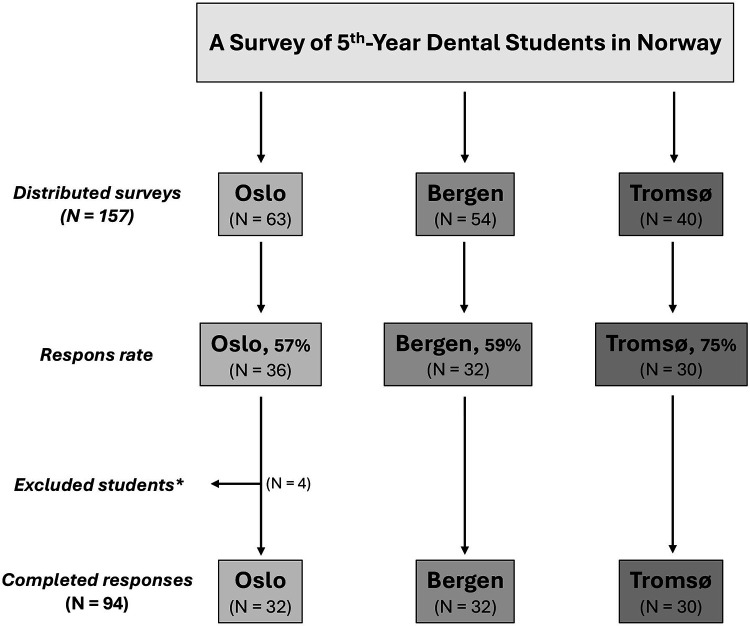
Flowchart of the survey among final-year dental students at the three dental schools in Norway (Oslo, Bergen, and Tromsø). The chart illustrates the number of students invited, the number of responses collected, and the final sample included in the study. *Indicates students who did not complete their entire dental education at the same institution.

This study employed a cross-sectional, questionnaire-based survey design and was conducted among final-year dental students enrolled at Norway's three dental schools: the University of Oslo (UiO), the University of Bergen (UiB), and the UiT Arctic University of Norway in Tromsø (UiT). The survey was distributed between September and December 2024. In total, 157 final-year students were eligible to participate (UiO: 63; UiB: 54; UiT: 40). An overview of the participant distribution, response rate, and the final number of included respondents is presented in the study flowchart (Figure 1). All three institutions offer a five-year dental program; however, they differ somewhat in how the basic sciences and clinical training are structured and integrated, providing a meaningful basis for comparison. At UiO, the curriculum follows a more traditional structure in which basic sciences and clinical education are largely separated. For example, craniofacial anatomy and physiology are introduced toward the end of the second year, marking the students' first formal exposure to the dentition and craniofacial complex. In contrast, students at UiB and UiT are introduced to oral aspects of dentistry earlier in their education, with elements of oral biology included already in the first year of the program.

### Questionnaire

The questionnaire was developed by the author team based on previous literature on curriculum integration and student perceptions (7, 10, 14). The items were refined through internal discussion and subsequently piloted with a group of 20 four-year dental students at UiO, whose feedback informed the final wording and structure of the instrument. At UiO, students received standardized invitations and automated reminders via institutional email, and two members of the author team additionally encouraged participation. At UiB and UiT, selected faculty members likewise encouraged students to participate in order to support higher response rates. Content validity was strengthened through expert review and iterative refinement of all items, supplemented by cognitive feedback from the pilot group. Because several constructs were assessed using single-item measures, internal consistency metrics were not applicable; however, the structured development process ensured conceptual clarity and reliable interpretation across items.

Data were collected through an electronic questionnaire administered using *Nettskjema*, an internet-based software program developed by the UiO. The instrument was designed to capture final-year dental students' perspectives on the integration of basic medical sciences within their education and their application of these sciences in clinical training. To ensure a broad representation of student views, the survey was distributed to all eligible students at the three institutions. The questionnaire contained both closed- and open-ended items and addressed several thematic areas. These areas included students' prior awareness and expectations before entering dental school, their experiences with the timing, teaching, and integration of basic sciences, and their perceptions of how basic medical knowledge contributes to clinical reasoning and treatment planning. Additional items explored students' views on challenges in teaching these subjects, potential approaches to increase engagement, and the perceived importance of basic sciences across different dental specialties. Participation in the study was voluntary, and all students were informed about its purpose prior to completing the questionnaire. The online survey tool *Nettskjema* ensured full anonymity of all respondents.

The study was reviewed according to institutional guidelines and deemed exempt from formal ethical review, as it involved voluntary, anonymous survey participation. Completion of the questionnaire was interpreted as informed consent. Missing responses were handled using listwise deletion at the item level, meaning that only fully completed responses for each question were included in the corresponding analysis. Open-ended responses were not subjected to formal qualitative analysis but were reviewed descriptively to provide contextual support for the quantitative findings.

### Statistical analysis

All survey responses were exported from Nettskjema. A significance level of *p* < 0.05 was applied throughout. Statistical methods were selected based on the structure of each survey item. For questions with ordinal response options (e.g., Likert-scale items), ordinal logistic regression models were used to examine differences between the three universities. Group differences were assessed using likelihood ratio tests by comparing models with and without university as a categorical predictor. For items with nominal (non-ordered) categorical response alternatives, differences between universities were examined using Fisher's exact test.

## Results

This study was conducted in 2024 and included 157 fifth-year students from Norway's three dental schools UiO, UiB and UiT who were invited to participate (Oslo: 63, Bergen: 54, Tromsø: 40). Of these, 98 students completed the survey, corresponding to response rates of 57% in Oslo (*n* = 36), 59% in Bergen (*n* = 32), and 75% in Tromsø (*n* = 30) (Figure 1). Students who had not completed their entire dental education at the same institution were excluded (*n* = 4 from Oslo), yielding a final sample of 94 participants: Oslo (*n* = 32), Bergen (*n* = 32), and Tromsø (*n* = 30) (Figure 1).

**Figure 2:**
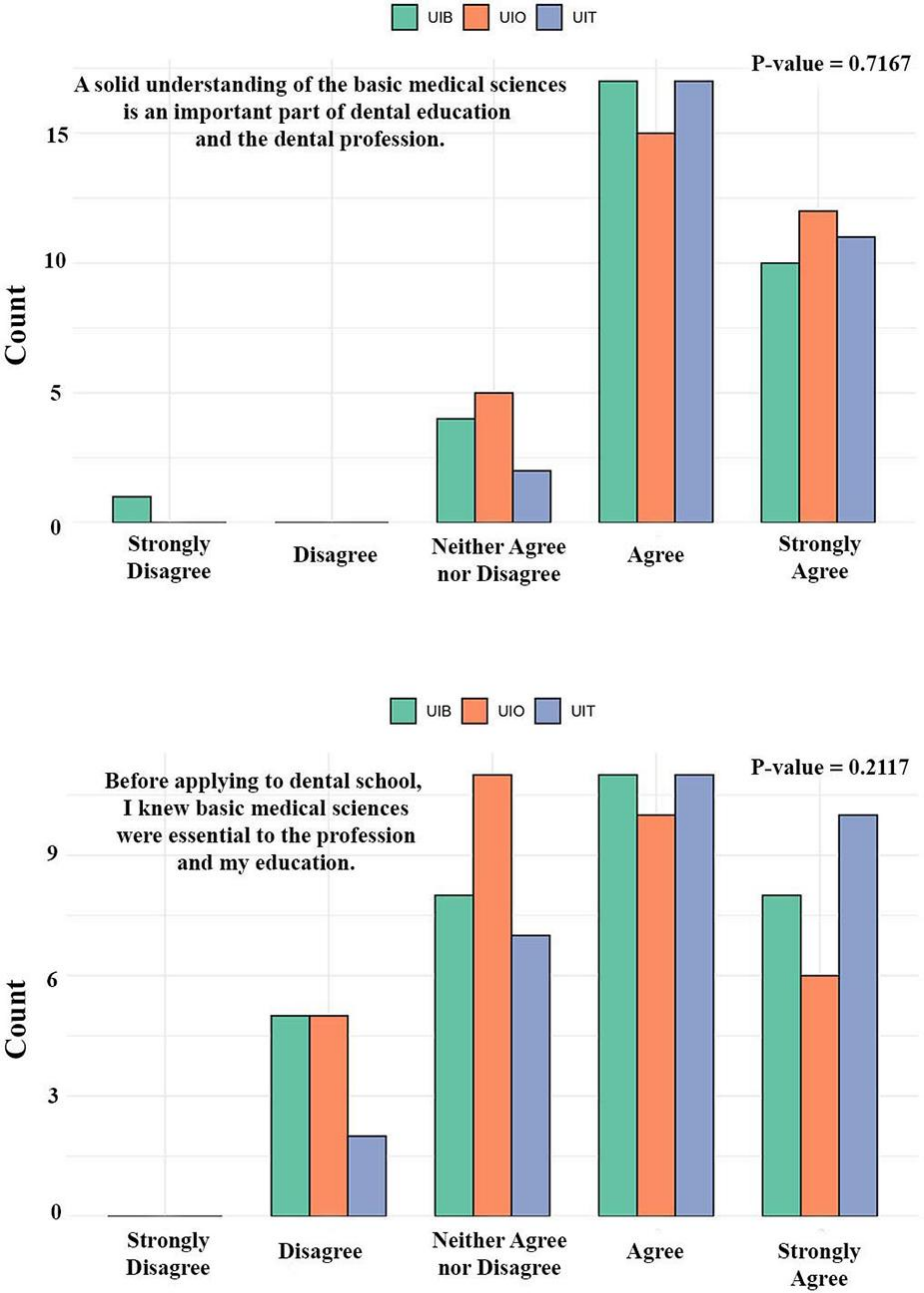
Insights from students at three dental schools in Norway regarding their prior awareness and expectations of the relevance of basic medical sciences before entering dental school. UiO, University of Oslo; UiB, University of Bergen; UiT, The Arctic University of Norway in Tromsø.

The first set of survey items explored students' prior awareness and expectations regarding the role of basic medical sciences in dentistry (Figure 2). Across all three institutions, most respondents emphasized that a solid understanding of basic medical sciences constitutes an essential component of dental education and professional practice. Furthermore, most students reported that they were already aware of the importance of these subjects before applying to dental school. There were no statistically significant differences in responses between the three universities (*p* > 0.05), indicating a shared recognition among students that basic medical sciences are foundational to dental training and the profession (Figure 2).

**Figure 3:**
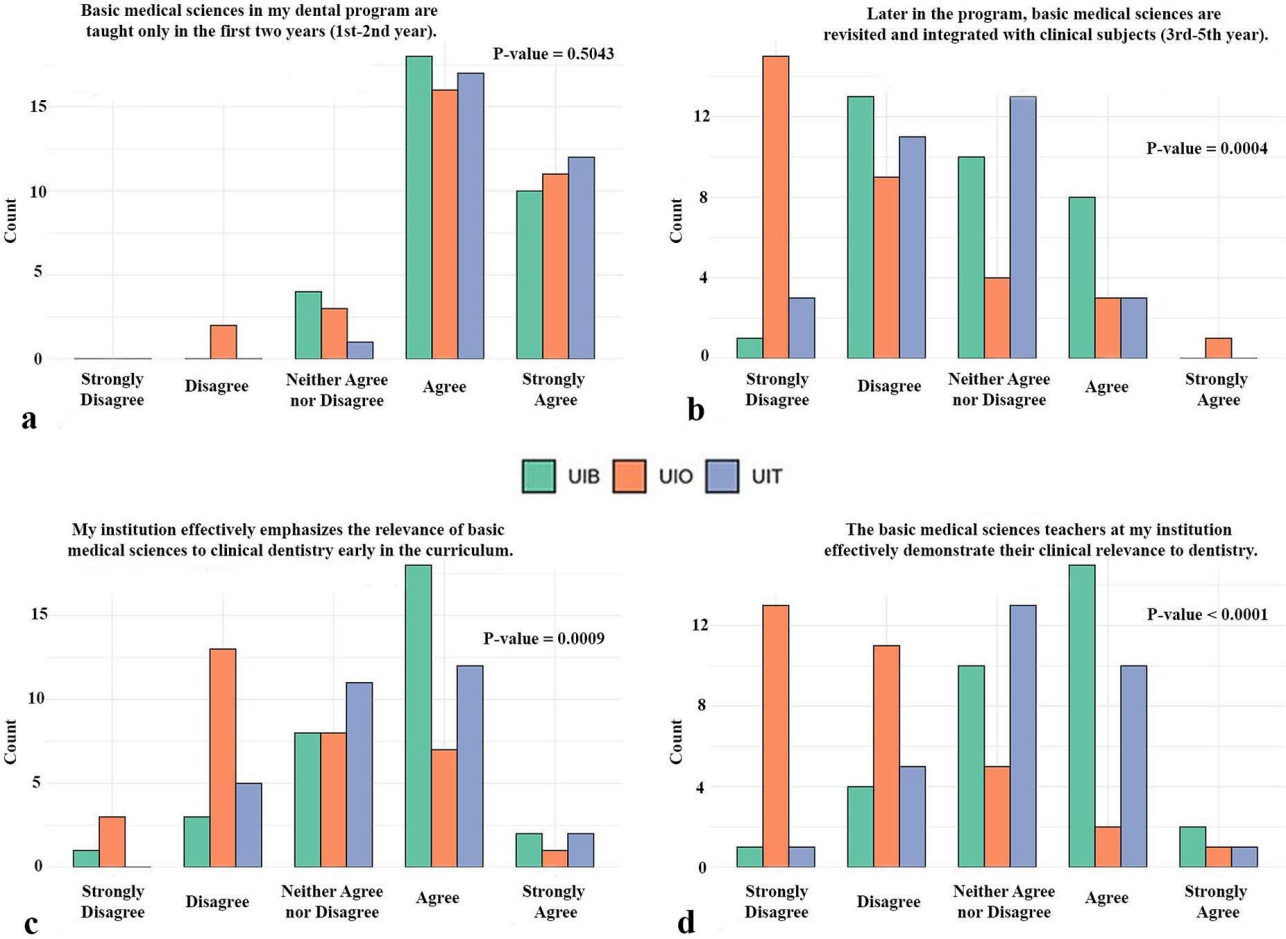
Experiences of students with the teaching of basic medical sciences. The figure illustrates their views on the timing and integration of these subjects within the curriculum, and the extent to which clinical relevance is emphasized by the institution and instructors. UiO, University of Oslo; UiB, University of Bergen; UiT, The Arctic University of Norway in Tromsø.

The second part of the survey examined how students experienced the teaching of basic medical sciences, including their timing within the curriculum, their integration with clinical subjects, and the extent to which their relevance to dentistry was emphasized (Figure 3). For the first item, students across all three institutions consistently reported that basic medical sciences are taught predominantly during the first two years of the program, with no significant difference observed between universities (*p* = 0.50) (Figure 3A). In contrast, notable differences emerged for the subsequent questions. Regarding the integration of basic medical sciences later in the curriculum, most students disagreed with this statement, yet the degree of disagreement varied significantly between institutions (*p* < 0.001). Students from the UiO expressed stronger disagreement compared with their peers at the UiB and UiT (Figure 3B). Similarly, significant institutional differences were evident in perceptions of whether the curriculum emphasizes the clinical relevance of basic sciences early in the program (*p* < 0.001). Students from UiB were more likely to agree, while those from UiO expressed the highest level of disagreement (Figure 3C). Finally, the same pattern was observed when students were asked whether teachers in basic medical sciences effectively demonstrate their clinical relevance to dentistry. Once again, students from UiO reported the lowest levels of agreement, while differences between institutions were highly significant (*p* < 0.0001) (Figure 3D).

**Figure 4:**
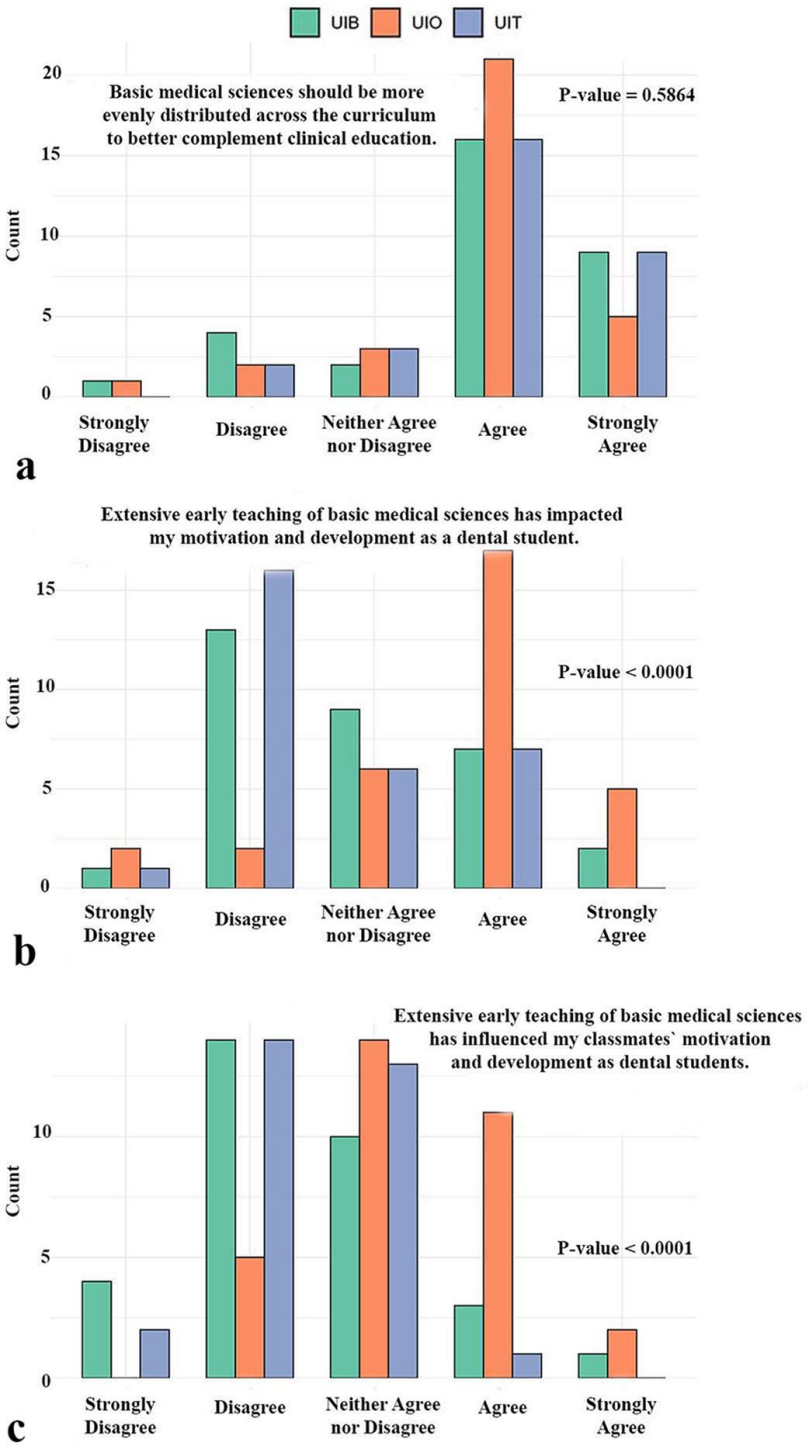
Perspectives of students on the structure and timing of basic medical sciences teaching. The figure highlights concerns that extensive front-loaded instruction may negatively influence their own and their peers’ motivation and development as dental students. It also reflects student views on the need for a more even distribution of basic sciences throughout the curriculum to better support clinical education. UiO, University of Oslo; UiB, University of Bergen; UiT, The Arctic University of Norway in Tromsø.

The next set of survey items addressed students' views on the structure and timing of basic medical sciences within the curriculum, with particular focus on whether the distribution of these subjects influences student motivation (Figure 4). A large majority of respondents across all three universities agreed or strongly agreed that basic medical sciences should be more evenly distributed throughout the dental program to complement clinical education better, with no significant differences observed between institutions (*p* = 0.59). However, clear contrasts emerged when students were asked about the impact of extensive early teaching of basic medical science on motivation. For their own motivation, responses differed significantly between institutions (*p* < 0.0001). At the UiO, approximately 70% of students reported that their motivation and development as dental students had been negatively affected, whereas most students at the UiB and UiT disagreed with this statement (Figure 4B). A similar pattern was observed when students were asked whether extensive early teaching had influenced the motivation of their classmates (*p* < 0.0001). While about 40% of UiO students reported that they believed their peers' motivation was also affected, students from UiB and UiT largely disagreed (Figure 4C).

**Figure 5:**
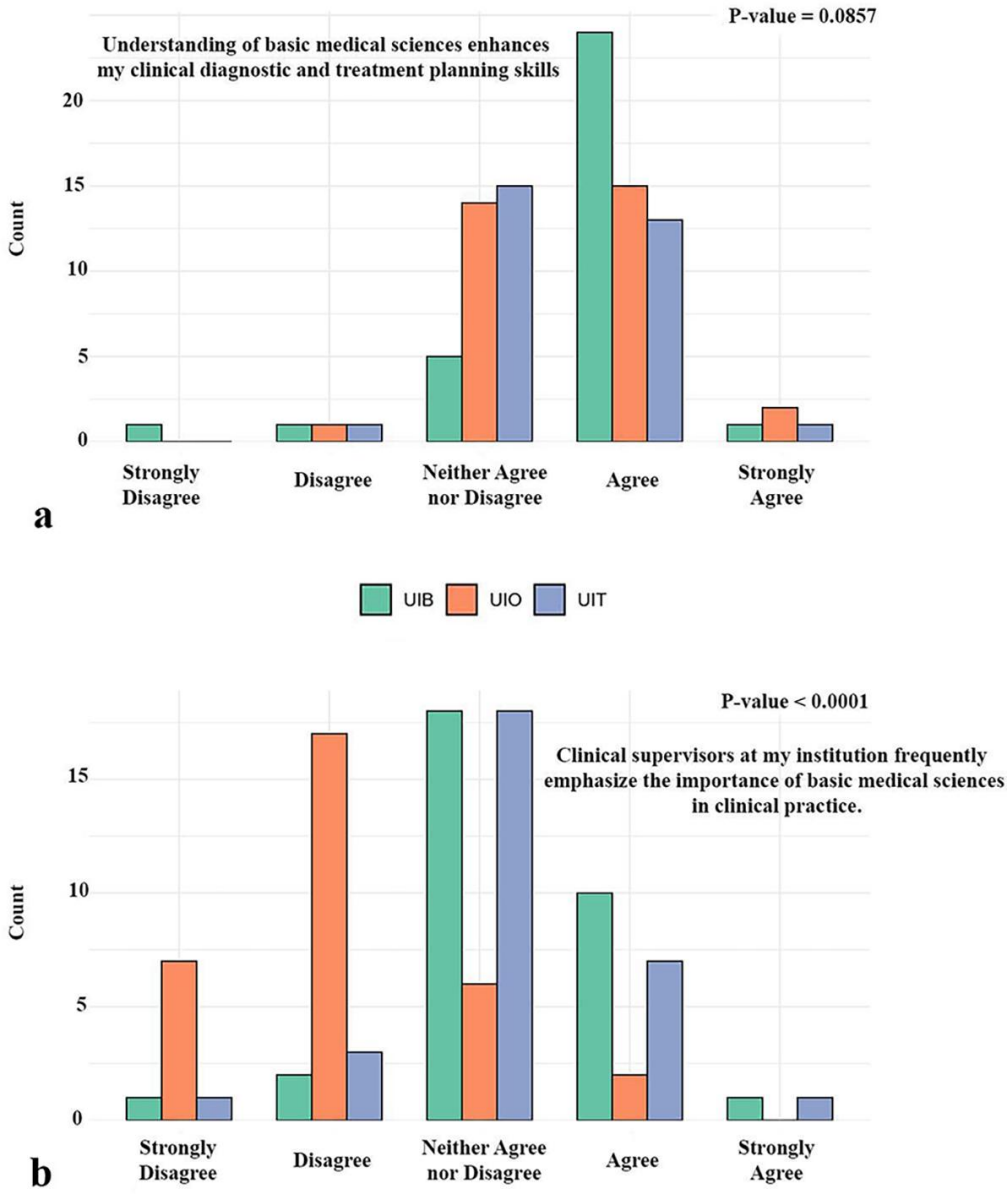
Student perspectives on the application of basic medical sciences in clinical settings. The figure illustrates how students perceive their understanding of basic sciences as supporting diagnostic and treatment planning, and how clinical supervisors emphasize the relevance of these subjects in dental practice. UiO, University of Oslo; UiB, University of Bergen; UiT, The Arctic University of Norway in Tromsø.

Our results further demonstrated how students perceive the application of basic medical sciences in clinical practice (Figure 5). When asked whether such knowledge contributes to diagnostic reasoning and treatment planning, about 55% of respondents across all three institutions agreed, while roughly 36% expressed a neutral position. Only a small minority disagreed, and no statistically significant differences were found between universities (*p* = 0.09) (Figure 5A). In contrast, a clear institutional difference emerged regarding the extent to which clinical supervisors emphasize the relevance of basic medical sciences in daily practice. While some students at the UiB and UiT reported agreement, the largest share at these institutions remained neutral. Students at the UiO, however, predominantly disagreed or strongly disagreed, indicating that they experienced relatively little emphasis from supervisors on the clinical importance of basic medical sciences (Figure 5B).

**Figure 6:**
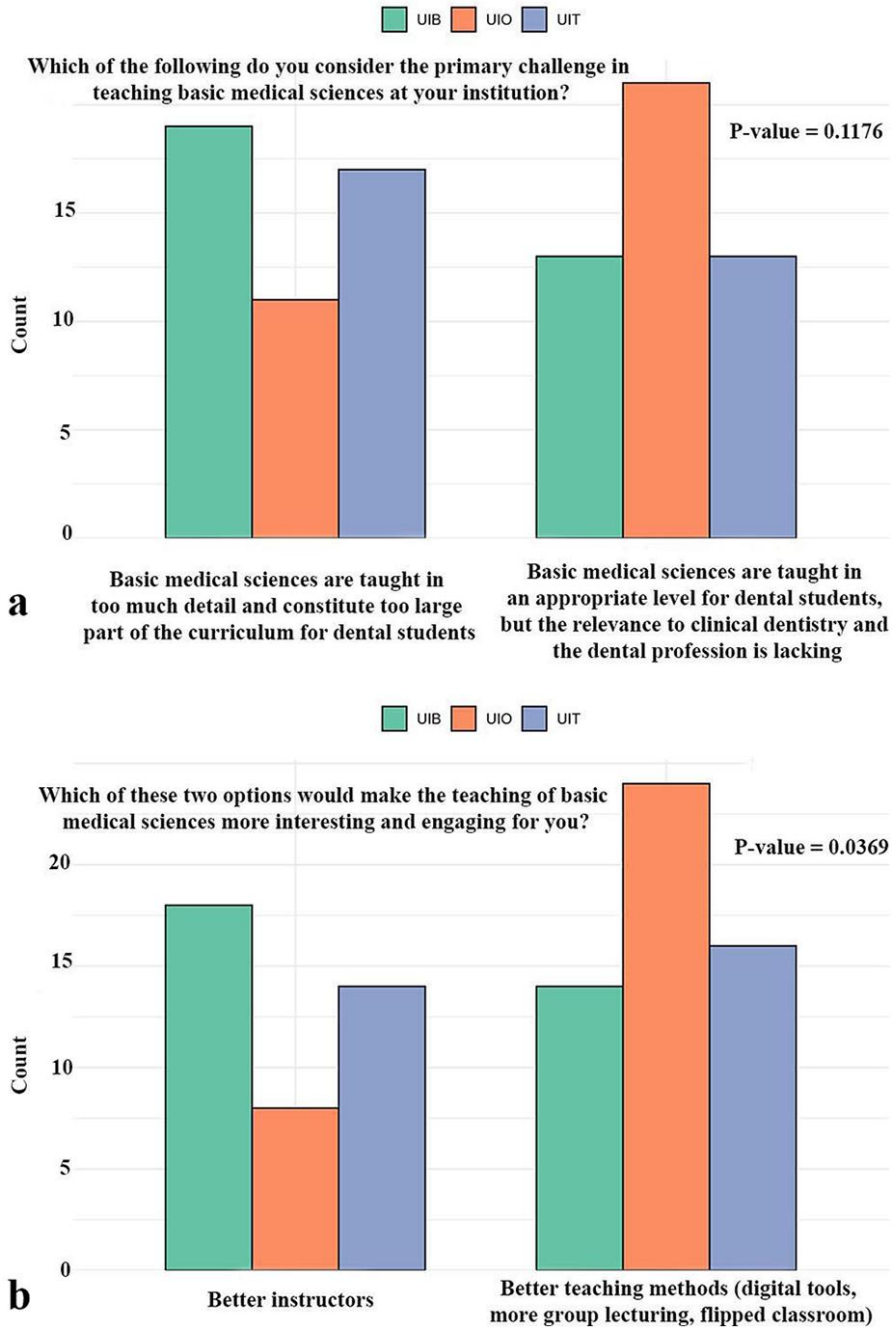
Student responses on the perceived challenges in teaching basic medical sciences, along with their views on potential approaches to make the subject more interesting and engaging. UiO, University of Oslo; UiB, University of Bergen; UiT, The Arctic University of Norway in Tromsø.

In addition, students were asked about challenges in the teaching of basic medical sciences and potential approaches that could make the subject more engaging (Figure 6). When considering the primary challenge, responses were divided between two main perspectives: that the basic sciences are taught in excessive detail and occupy too large a proportion of the curriculum, or that, while taught at an appropriate level, their clinical relevance is insufficiently emphasized. Students from all three universities expressed both views, with no statistically significant differences observed between institutions (*p* = 0.18) (Figure 6A). In contrast, significant variation emerged in how students evaluated potential strategies to improve teaching (*p* < 0.05). At the UiO, the majority favored improved teaching methods, such as the use of digital tools, more group-based learning, and flipped-classroom approaches. Students from the UiB and UiT, however, were more likely to emphasize the importance of higher quality instruction as the most effective means of making the subject more interesting and engaging (Figure 6B).

Students were also asked to evaluate the importance of basic medical sciences for different dental specialties (Table 1). Across all specialties, most respondents rated knowledge of basic medical sciences as either *important* or *very important*, with relatively few students selecting the lower categories. For endodontics, periodontology, pediatric dentistry, and orthodontics, perceptions were largely consistent across universities, with no significant differences observed (all *p* > 0.05). In contrast, notable variation emerged in responses regarding prosthodontics (*p* < 0.0001) and maxillofacial radiology (*p* < 0.05), where students differed in how strongly they emphasized the importance of basic sciences. Oral surgery and oral medicine also showed a trend toward variation between institutions, although this difference did not reach statistical significance (*p* = 0.09). When considering the total responses across all specialties combined, most students agreed that basic medical sciences play an essential role in specialization, and significant differences were observed between universities (*p* < 0.0001).

**Table 1:** Final-year dental students’ perceptions of the importance of knowledge in basic medical sciences for different dental specialties.

Dental specialties	Not important			Slightly important			Somewhat important			Important			Very important			*p*-value
	UIB	UIO	UIT	UIB	UIO	UIT	UIB	UIO	UIT	UIB	UIO	UIT	UIB	UIO	UIT	
Endodontics	0	0	0	1	4	0	8	8	12	16	16	17	6	4	1	0.3448
Periodontology	0	0	0	1	2	1	7	5	7	15	18	20	9	7	2	0.3778
Prosthodontics	1	7	0	17	13	5	14	7	16	9	4	9	0	1	0	7e-04
Pediatric dentistry	1	2	0	3	4	1	14	10	11	11	12	15	3	4	2	0.5443
Orthodontics	2	3	0	9	5	5	8	14	14	9	7	11	3	1	0	0.6091
Maxillofacial and oral radiology	1	7	0	4	10	2	13	8	15	11	3	11	2	4	2	0.0014
Oral surgery and oral medicine	0	1	0	0	0	0	1	0	1	14	13	20	17	18	9	0.0989
Total	5	20	0	35	38	14	65	52	76	85	73	103	40	39	16	<1e-06

The survey item asked: “*At some point in your career, you may choose to specialize in a particular field of dentistry. How important is knowledge of basic medical sciences for the various dental specialties?”* Reported p-values indicate whether significant differences were observed between responses from the three universities. UiO, University of Oslo; UiB, University of Bergen; UiT, The Arctic University of Norway in Tromsø.

## Discussion

This study explored how final-year dental students at Norway's three dental schools perceive the integration of basic medical sciences within their education and its relevance to clinical training and professional practice. By capturing student experiences across institutions with different curricular structures, the findings provide valuable insights into how curriculum design shapes perceptions of motivation, clinical applicability, and the overall role of basic sciences in dentistry.

A notable finding was that students across all three universities reported a strong awareness, even prior to admission, that basic medical sciences represent an essential part of dental education and practice. This suggests that prospective students recognize the centrality of biomedical knowledge in dentistry, likely reflecting broader societal and educational perspectives that regard the field as both a medical and technical discipline. The absence of significant institutional differences indicates that this awareness is broadly shared across the student population, independent of curriculum structure. From an educational standpoint, this early awareness underscores that dental students enter their programs with an expectation of biomedical relevance, which aligns with observations in other health professions where foundational sciences are universally acknowledged as central to clinical proficiency (6, 17, 18).

When evaluating their program structure, students consistently reported that most basic sciences are taught in the first two years, largely separated from clinical training, a traditional model still prevalent in many curricula (6). However, important differences emerged beyond this period. Students at the UiO, which follows a compartmentalized model, reported significantly less integration of basic sciences in subsequent years compared to students at UiB and UiT, where oral biology and clinical context are introduced earlier. This aligns with findings demonstrating that vertical integration, revisiting basic science in clinical years, enhances retention and clinical relevance (9, 10, 19).

Students at UiO consistently disagreed that their programs or faculty emphasize clinical applicability, while respondents at UiB and UiT reported greater acknowledgment of relevance.

These findings reflect evidence from integration frameworks suggesting that students learn and retain biomedical concepts more effectively when educators explicitly link these to clinical cases, symptoms, and decision-making processes (20–22). The absence of explicit links between theory and practice in separated models may foster perceptions of redundancy, potentially undermining student engagement and motivation (23). Indeed, student motivation varied significantly by institution: approximately 70% at UiO reported that the front-loaded basic sciences negatively affected their motivation, whereas students at UiB and UiT generally disagreed with this assessment. This finding echoes the curricular concern of “front-loading” theory, which has been linked to poor retention and integration when not accompanied by clinical framing. It also reflects broader evidence that early clinical experience supports motivation and facilitates the transition to patient-based learning (24, 25).

Regarding the perceived application of basic sciences, just over half of all students agreed that biomedical knowledge contributes to diagnostic reasoning and treatment planning, although a large minority adopted a neutral position. This finding suggests that while students conceptually value biomedical sciences, their ability to connect early theoretical learning to clinical reasoning may depend heavily on how frequently those connections are made explicit in teaching and supervision (26).

When students were asked to identify challenges in the teaching of basic sciences, two main perspectives emerged: that these subjects occupy too large proportion of the curriculum and are delivered with excessive detail, or that while the level of instruction is appropriate, clinical relevance is lacking. Both viewpoints were present across institutions, with no significant differences, indicating that these are common challenges in dental education (2, 27, 28). More revealing were the differences in how students evaluated strategies to improve teaching. Students at UiO most strongly supported the introduction of new pedagogical methods, such as digital tools, group-based learning, and flipped classrooms, reflecting a desire for innovation that might address the lack of perceived integration in their current curriculum. By contrast, students at UiB and UiT emphasized the importance of better instructors as the key to making basic sciences more engaging. This suggests that students in less integrated curricula may seek compensatory teaching strategies, whereas those in more integrated settings perceive pedagogical quality as the central determinant of engagement (6, 28).

When asked to consider the role of basic sciences in dental specialties, students overwhelmingly regarded biomedical knowledge as important or very important across all areas, from endodontics and periodontology to oral surgery. This consensus demonstrates that despite challenges in curricular design, students strongly recognize the lasting professional value of biomedical knowledge (29). The fact that significant differences emerged for prosthodontics and maxillofacial radiology may reflect variations in how these specialties are taught or emphasized across institutions, though overall agreement remained high. Importantly, this finding underscores that student dissatisfaction with teaching methods or curriculum structure should not be misinterpreted as a rejection of biomedical relevance. Rather, the challenge lies in ensuring that curricular design and teaching practices align with students' recognition of the importance of these subjects and support their ability to apply them effectively.

Taken together, these results illustrate how curriculum design profoundly influences student motivation, engagement, and perceptions of relevance (30). UiO's compartmentalized model appears to hinder motivation and integration, while UiB and UiT's early inclusion of clinical context supports more positive student experiences. This resonates with international trends toward integrated curricula to foster long-term retention, engagement, and clinical competence (6, 18, 26). From an educational policy standpoint, this suggests that integrated planning, distributing basic sciences over the full program, embedding clinical relevance early, and ensuring clinical faculty reinforce foundational concepts, may enhance student engagement and application. Pedagogical strategies such as case-based learning, team teaching, and active learning methods could also play key roles, as recommended by broader literature on health professions education (20).

Recruitment procedures varied somewhat between institutions and may represent a potential source of selection bias. Although all students received identical email invitations and no incentives were provided, the degree of personal encouragement differed. At UiO, invitations and reminders were automated, but two of the authors also encouraged students during ordinary academic interactions. At UiB and UiT, selected faculty members similarly encouraged participation in addition to the standardized email distribution. These differences, while moderate, may have influenced students' willingness to participate and contributed to variation in response rates. Consequently, institutional comparisons should be interpreted with some caution, as observed differences may partly reflect differential engagement or perceived expectations rather than solely underlying curricular structures.

Despite its strengths, including comprehensive national sampling and the opportunity to compare perceptions across institutions with distinct curricular structures, this study has some limitations. First, the cross-sectional design limits causal inference and captures perceptions only at a single point in students' education, preventing evaluation of how attitudes toward integration evolve over time or relate to later clinical competence. Second, the study relies on self-reported perceptions, which while valuable for understanding motivation and engagement, may not reflect actual knowledge retention, examination performance, or clinical application. Third, the absence of objective curriculum mapping or parallel faculty perspectives limits our ability to triangulate student views with structural curricular features or teaching practices. Fourth, although students were intentionally allowed to interpret the concept of “integration” in their own terms to capture diverse experiences, this approach introduces interpretive variability that may complicate comparisons across institutions. Finally, while response rates were high, the absolute sample size remains modest due to the small size of the national cohorts, which may reduce statistical power to detect smaller differences. Future research using longitudinal designs, objective performance data, curriculum audits, and multi-perspective input from both students and faculty would provide a more comprehensive understanding of how integration influences learning, motivation, and clinical readiness.

## Conclusions

This study demonstrates that while Norwegian dental students uniformly recognize the importance of basic medical sciences, their experiences of teaching, integration, and motivational impact vary substantially depending on curricular structure. Students at the University of Oslo, where preclinical and clinical phases are more distinctly separated, reported weaker vertical integration, lower emphasis on clinical relevance, and reduced motivation. In contrast, students at the Universities of Bergen and Tromsø, where oral and clinical elements are introduced earlier, reported more positive perceptions of relevance, engagement, and supportive reinforcement from instructors.

These findings highlight the critical role of curricular integration, particularly *vertical integration*, in sustaining motivation and supporting the application of biomedical concepts to clinical reasoning. Embedding biomedical content throughout the program, reinforcing its clinical applicability, and ensuring alignment between preclinical and clinical teaching may help strengthen knowledge retention and professional identity formation.

While the study offers valuable national-level insights, its cross-sectional design and reliance on student self-perceptions limit causal inference. Future research incorporating longitudinal designs, objective curriculum mapping, and multi-perspective data (e.g., faculty views, performance outcomes) may further elucidate how integration shapes student learning and clinical competence.

Overall, the results underscore the importance of deliberate, continuous integration of basic and clinical sciences in dental education to enhance relevance, motivation, and preparedness for modern clinical practice.

## Data Availability

The raw data supporting the conclusions of this article will be made available by the authors, without undue reservation.
