# Supplementary material for: Student perspectives on integrating basic and clinical dental sciences: insights from three dental schools in Norway
Source: Front Dent Med. 2026 Jan 26;7:1746468. doi: 10.3389/fdmed.2026.1746468 (PMC12883767; doi:10.3389/fdmed.2026.1746468)
Supplement: Supplementary file 1 [file Datasheet1.pdf]

The Importance of Basic Medical Sciences in Clinical Dentistry

Most dental education institutions recognize that foundational biomedical knowledge is a fundamental component of dental training.

Accordingly, dental curricula around the world are designed to ensure that students acquire essential basic medical science competencies and develop a solid understanding of subjects such as cell biology, genetics, anatomy, physiology, microbiology, immunology, and pathology.

We would like to learn how you experience, or have experienced, the teaching of basic medical sciences at your institution, and how you perceive their relevance for clinical dentistry and the dental profession.

I am a final-year dental student at the following institution:

	UiO
	UiB
	UiT

Have you completed your entire dental education at the same institution?

Yes	No

If no: Where and what have you studied previously? Please be as specific as possible.

Part 1:

Please evaluate the following statements on a scale from "**Strongly agree**" to "**Strongly disagree**".

A solid understanding of the basic medical sciences is an important part of dental education and the dental profession.

Strongly Disagree	Disagree	Neither Agree nor Disagree	Agree	Strongly agree

Before applying to dental school, I knew basic medical sciences were essential to the profession and my education.

Strongly Disagree	Disagree	Neither Agree nor Disagree	Agree	Strongly agree

Basic medical sciences in my dental program are taught only in the first two years (1st-2nd year).

Strongly Disagree	Disagree	Neither Agree nor Disagree	Agree	Strongly agree

Later in the program, basic medical sciences are revisited and integrated with clinical subjects (3rd-5th year).

Strongly Disagree	Disagree	Neither Agree nor Disagree	Agree	Strongly agree

My institution effectively emphasizes the relevance of basic medical sciences to clinical dentistry early in the curriculum.

Strongly Disagree	Disagree	Neither Agree nor Disagree	Agree	Strongly agree

The basic medical sciences teachers at my institution effectively demonstrate their clinical relevance to dentistry.

Strongly Disagree	Disagree	Neither Agree nor Disagree	Agree	Strongly agree

Basic medical sciences should be more evenly distributed across the curriculum to better complement clinical education.

Strongly Disagree	Disagree	Neither Agree nor Disagree	Agree	Strongly agree

Extensive early teaching of basic medical sciences has impacted my motivation and development as a dental student.

Strongly Disagree	Disagree	Neither Agree nor Disagree	Agree	Strongly agree

Extensive early teaching of basic medical sciences has influenced my classmates` motivation and development as dental students.

Strongly Disagree	Disagree	Neither Agree nor Disagree	Agree	Strongly agree

Understanding of basic medical sciences enhances my clinical diagnostic and treatment planning skills.

Strongly Disagree	Disagree	Neither Agree nor Disagree	Agree	Strongly agree

Clinical supervisors at my institution frequently emphasize the importance of basic medical sciences in clinical practice.

Strongly Disagree	Disagree	Neither Agree nor Disagree	Agree	Strongly agree

What do you consider to be the greatest challenge related to the teaching of basic medical sciences in the dental program at your institution?

Please select only one option.

	Basic medical sciences are taught in ***too much detail and constitute too large part of the curriculum*** for dental students.
	Basic medical sciences are taught in an appropriate level for dental students, but ***the relevance to clinical dentistry and the dental profession is lacking***.

What would make the teaching of basic medical sciences more engaging for you?

Please select only one option.

	Better teaching methods (digital tools, more group lecturing, flipped classroom)
	Better instructors

Do you have any suggestions for how the teaching of basic medical sciences at your institution could be improved to help educate the best possible dentists for the future?

<u>Part 2</u>

You are soon to graduate as a dentist.

You may at some point in your career wish to pursue one of the seven dental specialty programs.

Please assess the degree of importance, on a scale from ***"Not important"*** to ***"Very important"***, of basic medical science knowledge for each of the dental specialties listed below.

	**Not important**	**Slightly important**	**Somewhat important**	**Important**	**Very important**
Endodontics					
Periodontology					
Prosthodontics					
Pediatric dentistry					
Orthodontics					
Maxillofacial and oral radiology					
Oral surgery and oral medicine					
